# From its roots, organic inspires science, and vice-versa: science forum of the Organic World Congress 2020 in Rennes, France

**DOI:** 10.1007/s13165-020-00338-8

**Published:** 2020-12-08

**Authors:** Gerold Rahmann, Khalid Azim, Mohammad Reza Ardakani, Veronique Chable, Felix Heckendorn, Paola Migliorini, Bram Moeskops, Daniel Neuhoff, Frederic Rey, Ewa Rembiałkowska, Jessica Shade, Marc Tchamitchian

**Affiliations:** 1 Thünen-Institute, Trenthorst 32, 23847 Westerau, Germany; 2 Institut National pour la Recherche Agronomique (INRA), Rabat, Morocco; 3 Azad University, Tehran, Iran; 4 Agroecology Europe, Rome, Italy; 5 INRA, Paris, France; 6 FiBL, Frick, Switzerland; 7 FiBL, Eurre, France; 8 TP Organics, Brussels, Belgium; 9 University of Bonn, Bonn, Germany; 10 ITAB, Paris, France; 11 WULS, Warsaw, Poland; 12 The Organic Center, Washington DC, USA

## Introduction

The 6th ISOFAR Conference of Organic Agricultural Science of the Organic World Congress, co-organized with INRAe, FiBL, Agroecology Europe, TP Organics, and ITAB, was scheduled for September 2020. However, because of the COVID-19 pandemic, it was postponed to September 2021. Despite this postponement, the Scientific Board decided to produce an early special issue of *Organic Agriculture* with a selection of 15 accepted papers for publication prior to the 2021 conference date.

## Background of the Scientific Forum of the Organic World Congress 2020

The Organic World Congress 2020 has announced following introduction as call for papers for the scientific track (IFOAM 2019): “The scientific roots of organic systems are anchored in the four principles of IFOAM – Organics International: ecology, health, fairness, and care. Scientific research and evidence-based approaches are needed for the continuous improvement of organic food, systems, and culture. The Scientific Forum of the Organic World Congress 2020 will leverage this need by focusing on the following five themes, with a special focus on interdisciplinary research:

1. Ecological approaches to System Health
2. Product and Process Quality in Organic Agriculture: Methods and Challenges
3. Transitioning Towards Organic and Sustainable Food Systems
4. Innovation in Organic Farming: “Thinking Outside of the Box”
5. Political and Economic Frameworks as Drivers for a Vibrant Development of the Organic Sector”

## Paper selection

The call for papers was released in spring 2019, resulting in 315 submissions from 40 countries (Table 1). Over 200 qualified reviewers reviewed the submissions in the context of the 5 themes in order to assess their scientific quality and their ability to stimulate scientific discussion (Table 2). Currently, 98 papers have been accepted for presentation at the Organic World Congress (Table 3). Fifteen of those accepted papers are published here.

**Table 1:** Submitted and accepted papers for the Organic World Congress 2020 by country

Country	Submitted	OWC accepted	ORGA accepted
Argentina	1	1	
Austria	6	5	1
Bangladesh	4		
Belgium	1		
Brazil	4		
Cameroon	2	1	
Canada	8	4	
China	3		
Czech Republic	1		
Denmark	10	3	
France	76	21	3
Germany	44	17	2
Ghana	1		
Greece	1	1	
Hong Kong	1	1	
Hungary	5	2	
India	7	2	
Indonesia	1	1	
Italy	21	5	
Japan	3		
Kenya	2	1	
Luxembourg	6	4	1
Mexico	1		
Netherlands	4	2	
Nigeria	11	5	1
Norway	5	1	1
Peru	2		
Poland	8	2	1
Portugal	4	1	
Romania	2	1	
Serbia	3	1	
Spain	10	2	1
Sweden	4	1	
Switzerland	27	8	1
Taiwan	1		
Tunisia	3	1	1
Turkey	2		
Ukraine	2		
Great Britain	6		
USA	11	4	2
Not specified	1		
Total	315	98	15

**Table 2:** Submitted and accepted papers for the Organic World Congress 2020 by topic

Thematic themes	Total	OWC accepted	ORGA accepted
• Ecological approaches to systems’ health	82	29	2
• Product and process quality in Organic Agriculture: methods and challenges	40	11	6
• Transition towards organic and sustainable food systems	104	32	1
• Innovation in Organic farming: “thinking out of the box”	63	20	4
• Political and economic frameworks as drivers for a vibrant development of the organic sector	26	6	2
Total	315	98	15

**Table 3:** Submitted and accepted papers for the Organic World Congress 2020 by subject

Scientific subject	Total	OWC accepted	ORGA accepted
• Agropolicy, macroeconomy, sociology and research	45	10	1
• Animal health	4	2	
• Animal husbandry non-ruminants	12	4	1
• Animal husbandry ruminants	17	4	
• Arable crops	16	8	1
• Crop protection	17	7	3
• Cropping systems and soil tillage	28	14	2
• Food processing	8		
• Food quality and nutrition	20	5	2
• Horticulture, permanent crops and agroforestry	21	4	
• Market, consumers and certification	27	6	1
• Nutrient management	32	11	1
• Plant breeding	26	8	3
• Resource conservation and sustainability	27	7	
• Soil fertility	10	5	
• Weed management	5	3	
Total	315	98	15

The selected papers in this special issue show the broad spectrum of Organic Agricultural research and highlight concepts and ideas for discussion. Scientific discussions are critical for the development of research, not only in advancing knowledge and methodology but also in expanding innovation in approaches and visions. These discussions will provide the scaffolding for the 6th ISOFAR Conference of Organic Agricultural Science of the Organic World Congress in Rennes in 2021.
